# What Does Standard 5.8 Measure? Nodal Adequacy as an Accountability Metric in Lung Cancer Surgery

**DOI:** 10.3390/medicina62101848

**Published:** 2026-09-24

**Authors:** Vasiliki Androutsopoulou, Prokopis-Andreas Zotos, Namariq Abbaker, Marco Scarci, Ugo Cioffi, Fabrizio Minervini, Dimitrios E. Magouliotis

**Affiliations:** 1 Department of Cardiothoracic Surgery, University of Thessaly, Biopolis, 41110 Larissa, Greece; androutsopoulouvasiliki@uth.gr (V.A.); zotospro@hotmail.com (P.-A.Z.); 2 Department of Cardiothoracic Surgery, Hammersmith Hospital, Imperial College Healthcare, National Health Service (NHS) Trust, London W2 1NY, UK; n.abbaker@nhs.net (N.A.); marco.scarci@nhs.net (M.S.); 3 Department of Surgery, University of Milan, 20157 Milan, Italy; ugo.cioffi@guest.unimi.it; 4 Department of Thoracic Surgery, Luzern Kanton Hospital, 6000 Luzern, Switzerland; fabrizio.minervini@luks.ch; 5 Department of Cardiac Surgery Research, Lankenau Institute for Medical Research, Wynnewood, PA 19096, USA

**Keywords:** non-small cell lung cancer, lung cancer, lymph node staging, lymphadenectomy, quality metrics, Commission on Cancer, accreditation, sublobar resection, quality improvement, thoracic surgery

## Abstract

This unstructured narrative review appraises lymph node evaluation as an accountability measure in non-small cell lung cancer surgery. Commission on Cancer Operative Standard 5.8 requires assessment of at least three mediastinal and one hilar nodal station for every curative-intent resection, with accreditation consequences. We ask what the standard has been validated to measure, and at which level of attribution. Neither the count-based nor the station-based threshold was derived from interventional evidence. The reported value reflects surgeon technique, surgeon-led specimen process, pathologist effort, operative platform, and case mix together, which bears on attribution to individual surgeons rather than on the legitimacy of the measure. We separate two causal models linking nodal evaluation to survival and examine each against evidence identified by a reproducible search, including the biomarker-directed and neoadjuvant eras and sublobar resection. Anchored to the National Quality Forum and Donabedian criteria, we conclude that Standard 5.8 is well-supported as an institutional minimum standard for staging completeness, that the evidence identified does not yet support extension to individual-surgeon benchmarking, that compliance should be reported stratified by resection type, and that low sublobar compliance most plausibly identifies a real quality problem. Nothing here reduces the obligation to perform systematic nodal assessment.

## 1. Introduction

Few process measures in surgical oncology have traveled as far or as quickly as the pathological evaluation of regional lymph nodes in lung cancer. Within roughly two decades, it has moved from an operative preference debated among thoracic surgeons, to a recommendation in professional guidelines, to a surveillance metric, and finally to an accreditation requirement with institutional consequences for non-compliance [1,2]. That trajectory is unusual, although not unique. Several surgical process measures have acquired regulatory force on the strength of observational associations before interventional confirmation, and the Surgical Care Improvement Project measures are the best-documented example: publicly reported adherence to individual measures was not associated with lower infection rates when tested at scale [3]. Nodal evaluation acquired its status largely on the strength of observational associations between the thoroughness of nodal examination and survival.

The American College of Surgeons Commission on Cancer (CoC) originally framed adequacy in terms of node count, requiring examination of at least ten lymph nodes retrieved from anywhere within the chest. That criterion was subsequently abandoned in favor of Operative Standard 5.8, which is defined anatomically: lymph nodes must be reported from at least three distinct mediastinal (N2) stations and at least one hilar (N1) station, for all curative-intent resections for lung malignancy, with synoptic documentation [4]. The shift was a genuine conceptual improvement, since a station-based criterion at least specifies where the surgeon must look. Adherence is now audited. Early site-review data indicated that a little over half of accredited programs met the standard, and a substantial national quality improvement collaborative has since been mounted to close the gap [5,6].

The response of the field to this compliance shortfall has been almost entirely operational. The wide array of literature now describes barriers to adherence, interventions that raise adherence, and institutional characteristics that predict adherence, and we have previously synthesized the literature and the guideline framework surrounding it [7]. The present review asks a different question. Compliance work presumes that the measure is valid for the purpose to which it is put and that the problem is implementation. Before accepting that premise, it is worth asking what Standard 5.8 measures, whom it holds accountable, and what claim about patient benefit its use as an accountability instrument actually licenses.

These are not rhetorical questions. A quality metric makes an implicit causal assertion: that the measured behavior is under the control of the party being held accountable, and that changing it changes outcomes for patients. If either limb fails, the metric may still be useful for standardizing documentation while nonetheless misattributing performance and, in the worst case, distorting clinical decisions at the margin. Others have noted that the field has been slow to settle on defensible benchmarks for lung cancer surgery quality [8]. Our objectives are therefore to establish what Standard 5.8 has been validated to measure and at what level of attribution, to state plainly what it has not been validated to measure, and to specify what further validation would require. The review is organized as follows. Section 2 sets out its scope, the way evidence was assembled, and the distinct questions the metric raises. Section 3 traces the provenance of the threshold. Section 4 examines what the reported value contains and what that implies for attribution. Section 5 examines the survival association, the causal models behind it, and the adjuvant and neoadjuvant landscape. Section 6 addresses sublobar resection. Section 7 examines alternatives and changing context. Section 8 evaluates the metric against an established measure–evaluation framework and states its clinical implications. Section 9 and Section 10 give limitations and conclusions.

## 2. Scope, Evidence Assembly, and the Questions Addressed

This is an unstructured narrative review and not a systematic review. It cannot, and does not, claim to establish that any metric is invalid, nor to characterize the totality of the validation evidence. What it can do is document the validation evidence we were able to identify through the reproducible search described below, and set out the conceptual distinctions that any future validation must respect. The claims made below are claims about what we did and did not identify, not claims that the undemonstrated is false.

The search was run in PubMed/MEDLINE on 12 September 2026, from database inception, using the following string: (lung cancer[tiab] OR lung neoplasms[MeSH]) AND (lymph node*[tiab] OR lymphadenectomy[tiab] OR nodal staging[tiab]) AND (quality metric[tiab] OR quality measure[tiab] OR quality standard[tiab] OR Commission on Cancer[tiab] OR compliance[tiab] OR adherence[tiab]) AND (survival[tiab] OR recurrence[tiab] OR upstaging[tiab]) AND (resection[tiab] OR lobectomy[tiab] OR segmentectomy[tiab]). It returned 49 records. Two authors screened titles and abstracts. Twenty-six were excluded: four case reports, five reviews without primary comparative data, five studies of other malignancies or small cell histology, and 12 addressing a different exposure or question (preoperative staging, prognostic nodal descriptors, operative technique, adjuvant therapy trials). Twenty-three primary studies were retained, of which six were already cited in the earlier version of this review, and 17 were newly identified [9,10,11,12,13,14,15,16,17,18,19,20,21,22,23,24]. Reference lists of retained studies and of the relevant guideline and consensus documents were then hand-searched, which supplied the randomized trial, the population-based comparisons, and the registry studies that predate the quality-metric vocabulary of the search string. No database other than PubMed/MEDLINE was searched; Embase and Web of Science were not available to the authors, and this is a limitation stated again in Section 9.

Studies were included in Table 1 and Table 2 if they met all of the following: they compared a defined nodal adequacy criterion (count-based, station-based, lobe-specific, or guideline-composite) against a patient-centered outcome (survival or recurrence) or against an intermediate on the causal path (nodal upstaging, adjuvant therapy receipt, perioperative morbidity); they used a randomized design or an adjusted observational design with a defined comparator; and they reported the criterion in a form that maps onto the thresholds under discussion. Where studies conflict, both are reported. No validated risk-of-bias instrument exists for this study type; instead, Table 2 carries for each study a structured appraisal of design, setting, adjustment, endpoint, and the principal threats to validity with their likely direction, so that the reader can see which conclusions rest on which evidence. The appraisal identifies two recurrent threats that pull in opposite directions: confounding by indication and institutional co-processes, which favor the metric, and contamination and ceiling effects at high-volume centers, which bias toward the null.

The metric raises several distinct questions that are easily run together, and we list them here so that each can be answered in its own place: whether three mediastinal stations is the optimal threshold (Section 3); whether lymphadenectomy itself has therapeutic benefit beyond adequate sampling (Section 5.2); whether more thorough staging improves survival (Section 5.3, Section 5.4, Section 5.5, Section 5.6 and Section 5.7); whether Standard 5.8 fairly measures an individual surgeon (Section 4); whether it fairly compares institutions (Section 4 and Section 8); whether it should influence accreditation (Section 8); and whether different specification is needed after sublobar resection (Section 6). A threshold may be imperfect without the underlying quality domain being invalid. A process measure may be useful without independently predicting overall survival. A measure may require case-mix adjustment without being worthless. A metric may be unsuitable for ranking individual surgeons while remaining useful for institutional quality improvement. We accept each of these propositions, and the argument that follows is constructed so that criticism of one does not masquerade as refutation of another.

### At Which Level Is Standard 5.8 Actually Used?

Because the conclusions below distinguish institutional from individual-surgeon use, it matters how the standard is actually applied. Its formal accreditation consequence is institutional: a Commission on Cancer program must meet the criterion in at least 80% of eligible resections, and site review operates at the program level [5,6]. We identified no formal use of Standard 5.8 for individual-surgeon credentialing, privileging, or sanction. Surgeon-level application does occur, in two forms. Single-institution quality programs report and compare compliance by individual surgeon [32], and regional collaboratives analyze concordance at the surgeon level, most explicitly in a statewide cohort of 3031 resections by 43 surgeons in which surgeon volume was an independent determinant of concordance [10], with surgeon-level feedback forming part of the collaborative improvement mechanism [33]. The individual-surgeon use of the metric is therefore real but informal and improvement-oriented, not formal and consequential. The scope of this review is accordingly narrower than the earlier version implied: the institutional accreditation use is the primary object of appraisal, and the surgeon-level discussion in Section 4 is directed at the emerging feedback and benchmarking use, and at the prospect of its extension, rather than at a formal accountability regime that does not yet exist.

## 3. Provenance of the Threshold

The ten-node criterion did not emerge from a trial. It emerged from a body of registry analyses showing that patients whose specimens yielded more examined nodes survived longer. In an institutional series of 442 stage I resections, greater node counts were associated with improved disease-free and overall survival [34], and in 16,800 node-negative patients in the Surveillance, Epidemiology, and End Results program, survival improved incrementally up to roughly 11 to 16 examined nodes, with no gain beyond [35]. The authors of the latter study attributed the association to reduction in staging error rather than to any therapeutic effect, and were explicitly reluctant to specify an optimal number. Combined with the reasonable intuition that a more thorough examination produces a more accurate stage, these associations became a threshold. The number itself was a convenient cut point in a continuous and noisy distribution rather than a biological inflection, and subsequent registry analyses did not support it as such: in 102,225 resections in the National Cancer Database, the survival association was concentrated between zero and roughly four or five examined nodes with no further gain beyond, and the authors concluded that the data did not justify a ten-node metric [18]; an institutional cohort with a SEER comparison found no survival difference between one and nine, ten, or more nodes [20]. Its most obvious defect was anatomical indifference: ten nodes were retrieved entirely from intrapulmonary and hilar tissue within a lobectomy specimen and satisfied the criterion without any mediastinal assessment whatsoever, and the lower-volume centers were substantially less likely to meet it even when they operated well in other respects [2].

Standard 5.8 corrected that specific defect by relocating adequacy from the count to the map. It did not, however, arrive with interventional support of its own. The station threshold of three mediastinal and one hilar was a codification of prevailing expert practice, consistent with the systematic sampling protocols used in earlier trials, rather than an independently validated cut point. The choice of three rather than two, or four, has no empirical derivation that can be traced to a comparative study; when the comparison has subsequently been made directly in a single high-volume institution, two and three stations have not separated on oncological outcome in early-stage disease [29].

It is also worth recording that this is a regional convention rather than a universal one. Japanese and other East Asian practice has historically emphasized systematic and lobe-specific nodal dissection defined by anatomical drainage patterns, whereas North American practice consolidated around counts and then stations. The International Association for the Study of Lung Cancer (IASLC) approached the same problem from a third direction, embedding nodal evaluation within a definition of complete resection that also requires free margins, absence of extracapsular extension, and a negative highest mediastinal node, and that explicitly admits a category of uncertain resection when the conditions cannot be verified [36,37]. Table 1 sets these frameworks alongside one another.

The comparison is instructive because the frameworks disagree not merely on where to place the threshold but on what the object of measurement is. Counts measure retrieval yield. Stations measure anatomical coverage. The IASLC definition measures procedural completeness. National composite criteria bundle nodal evaluation with margin status and resection extent, so that a single reported failure rate cannot distinguish an inadequate lymphadenectomy from a positive margin [1,38]. Three of these can be satisfied while another is violated. A field that has not agreed on what it is measuring should be cautious about the inferences it attaches to any particular measurement of it.

## 4. What the Metric Contains: An Attribution Problem

The central point of this section is about attribution, not legitimacy. The value reported under Standard 5.8 is a composite of several independently varying contributors (Figure 1). That does not make it an illegitimate measure of the nodal-assessment pathway. It does bear on which party the measure can fairly be used to hold accountable, and at what level. Three levels need to be kept apart. The first is individual technical performance: the extent and accuracy of the dissection in the chest. The second is surgeon-led process performance: station identification, separate retrieval and labeling, communication with pathology, and the surgeon’s part in establishing an institutional specimen pathway. The third is institutional performance: the pathway as a whole, including pathology, registry, and the systems that support the first two. Good thoracic oncological surgery includes the second level as well as the first, and the specimen-collection evidence reviewed below is as much a demonstration of that as it is of pathway effects. The attribution problem this section describes is the problem of reading a value that reflects all three levels as though it reflected only the first.

### 4.1. Surgeon Behavior

Surgeon-attributable variation is real and is the component the metric is intended to capture. In a single tertiary center applying Standard 5.8, compliance differed significantly across three surgeons, ranging from 70.9% to 93% [32]. Statewide collaborative data show that this component is genuinely modifiable: unblinded hospital-level feedback within a thoracic quality collaborative raised the proportion of lobectomies with five or more stations sampled from under 40% to roughly 80% over five years [33]. To the extent that a metric is meant to identify and correct a modifiable behavior, this is the evidence in its favor, and it is not trivial.

### 4.2. Pathologist Effort and Specimen Handling

The second contributor is invisible in the operating room. Node yield after lung cancer resection varies substantially by individual pathologist. In a single-institution analysis of 836 resections in which cases were matched to detailed pathologist reports, the individual pathologist was an independent determinant of node yield, with inter-pathologist coefficients spanning from approximately minus 5.6 to plus 11.3 nodes, and the individual pathologist also independently predicted the number of stations reported [39]. That range is wider than most of the differences between institutions that quality programs attempt to adjudicate. Two caveats apply to both this study and the surgeon-level compliance spread cited above. Each comes from a single institution, and neither is a formal variance-partitioning study; the proportion of total variance attributable to surgeon, pathologist, and institution has not been estimated in any dataset, and the generalizability of these point estimates is therefore unknown. The claim they support is that the contributions are separable and non-trivial, not that any particular share is established. A further source of variance sits between the two professions: the boundaries of several mediastinal stations, notably the division between stations 4 and 10 and the extent of station 7, are defined by anatomical landmarks that are applied with imperfect consistency, and the IASLC map that governs station assignment was itself introduced to reduce inter-observer disagreement in exactly this respect [40].

The specimen-handling pathway is equally consequential and, importantly, is separately modifiable. Deployment of a lymph node specimen collection kit, which prompts intraoperative separation and labeling of nodal material, has produced changes in a magnitude that surgeon education alone has not matched. In a population-based stepped-wedge comparison, non-examination of mediastinal nodes fell from 27% at baseline and 22% under a heightened-awareness intervention to 1% with the kit, while CoC benchmark attainment rose from 14% and 30% to 77% [28]. At institution level, the adjusted odds of attaining Operative Standard 5.8 with the kit were an order of magnitude higher than in non-implementing institutions [41].

This finding deserves attention because of what it implies about attribution, and we state the implication carefully. If a change in specimen packaging can move measured compliance from 14% to 77%, then a large part of what the metric was previously recording was determined between the operating table and the grossing bench. That does not weaken the relationship between the metric and surgical quality in the second of the three senses distinguished above. Identifying stations, retrieving tissue, labeling specimens separately, communicating with pathology, and leading the adoption of a collection protocol are surgeon-led process performances, and a metric that rewards them is measuring something a surgeon should own. What the kit data do show is that the metric is more sensitive to that process layer than to individual technical performance in the chest, and that comparing surgeons who work within different specimen pathways on the reported value is a comparison of pathways as much as of surgeons.

### 4.3. Case Mix

A further contributor is the patient. Compliance is significantly lower for non-adenocarcinoma histology, is influenced by laterality and operative approach, and falls sharply with decreasing resection extent [32]. Institutional characteristics behave in the same way, with adherence to nodal sampling measures tracking teaching status and volume in directions that are opposite to adherence to some care-coordination measures at the same institutions [38]. None of this is surgeon performance in any meaningful sense, yet all of it enters the reported figure unadjusted. Qualitative work with surgeons, pathologists, and data analysts implementing the standard found that participants broadly accepted the principle of a minimum threshold while remaining uncertain about the evidence supporting the particular one they were being held to, and identified these technical and clinical constraints precisely as barriers [42].

### 4.4. Operative Platform

The final contributor is the technology in the room. The evidence here is heterogeneous, and we present it as such, with effect estimates and their uncertainty where reported. A systematic review and meta-analysis of 25 studies comprising 50,404 patients found no significant difference in lymph node upstaging between robot-assisted and video-assisted approaches, odds ratio 0.89 (95% CI 0.76 to 1.04) [43]. A propensity-matched series of 613 patients found that the number of dissected nodal stations was significantly higher with the video-assisted approach, 5.9 versus 4.8, yet the proportion of patients upstaged after histopathological analysis was similar between groups [44]. A single-institution analysis of 226 patients with clinical N0 disease found the opposite direction on yield, with more nodes dissected robotically and a longer dissection time, and again no significant difference in the upstaging rate of the N factor [45]. A meta-analysis restricted to prospective studies found significantly more nodal stations dissected in the robotic arm, a mean difference of 1.07 stations, with no difference in the upstaging rate, odds ratio 0.67 (95% CI 0.36 to 1.25) [46].

The comparative series above are heterogeneous, and heterogeneity is not evidence of confounding. We therefore asked the narrower question a reviewer put to us: Does operative platform independently affect Standard 5.8 concordance after adjustment for surgeon, procedure, and case characteristics? We identified one analysis that addresses it. In a statewide collaborative cohort of 3031 resections by 43 surgeons, with surgeon volume, practice type, and resection type in the model, open resection (odds ratio 0.46, 95% CI 0.33 to 0.66) and video-assisted resection (0.35, 0.25 to 0.48) were each independently less likely than robotic resection to be concordant, while nodal upstaging did not differ between concordant and non-concordant groups [10]. In the National Society of Thoracic Surgeons database, resection type was a strong determinant of concordance and upstaging was higher in concordant cases, with no morbidity or mortality penalty [9]. One adjusted analysis is not a demonstrated confounder, and we have withdrawn the earlier recommendation that institutional comparisons adjust for platform mix. Platform is better described as a candidate stratification variable whose independent effect has been shown once and requires replication, and as a reminder that measured station yield can move without occult disease detection moving with it.

### 4.5. What the Attribution Problem Does and Does Not Imply

Modern lung cancer surgery is delivered by a pathway rather than by an individual, and the observation that multiple professionals influence a quality measure is not, by itself, evidence against the measure. It is evidence about the level at which the measure can be fairly applied. A measure determined by surgeon, pathologist, specimen protocol, platform, and case mix is a reasonable measure of an institution because institutions are responsible for all five. It is a much weaker measure of a surgeon because surgeons control one of them fully and share the others. The compliance literature has largely used Standard 5.8 at the institutional level, which is the level at which the attribution problem is least severe, and the collaborative improvements reported under it are real [6,33]. The concern arises when the same value is read back as a measure of individual surgical performance, which the structure of the metric does not support. Nothing in this section should be read as implying that surgeons are not responsible for nodal assessment. They are. The point is that a metric which cannot separate their contribution from the pathway’s should be used to improve the pathway, not to rank them.

## 5. The Survival Association and Its Interpretation

The case for nodal adequacy as a quality metric rests on its association with survival. That association is real and reproducible. The question is what generates it, and what evidentiary standard the answer should be held to.

### 5.1. Two Causal Models

Two mechanisms are routinely invoked, often within the same paragraph, although they have quite different implications (Figure 2). The staging-accuracy model holds that thorough nodal evaluation improves survival indirectly by detecting occult nodal disease, upstaging the patient, and thereby redirecting them to adjuvant systemic therapy that they would otherwise not receive. The therapeutic model holds that the dissection itself confers benefit by removing disease that would otherwise recur.

These are not interchangeable. Under the staging model, the benefit is contingent on the existence and uptake of effective adjuvant therapy, and the metric is a proxy for correct treatment allocation rather than for surgical skill. Under the therapeutic model, the benefit is intrinsic to the operation and the metric is closer to a genuine measure of surgical adequacy. Each generates different predictions, and both are testable. Neither model, it should be said, is what most thoracic surgeons mean when they describe the purpose of lymphadenectomy. The purpose is accurate pathological staging, which serves prognosis, the identification of occult disease, the determination of complete resection, and treatment planning together. Those purposes are not in question here. What is in question is the further inferential step from a staging-completeness standard to a validated measure of surgical quality, which requires one of the two models above to hold.

### 5.2. What Z0030 Tests and What It Does Not

ACOSOG Z0030 is the only randomized trial in this area, and it is frequently cited for more than it can support, including by ourselves in an earlier draft. Its design should be stated precisely. Patients with clinical T1 or T2, N0 or non-hilar N1 disease first underwent protocol-driven systematic sampling of specified stations (2R, 4R, 7, and 10R on the right; 5, 6, 7, and 10L on the left), a protocol that exceeds the requirements of Standard 5.8. Only if that sampling was negative on frozen section were they randomized to complete mediastinal dissection or no further dissection. Occult N2 disease was identified in 21 patients in the dissection arm, 4% of those randomized. At a median follow-up of 6.5 years, there was no difference in median survival, in five-year disease-free survival, or in local, regional, or distant recurrence [25]. The morbidity analysis found no significant difference in complication rates or mortality between arms; complete dissection added a median of 15 min of operative time and modestly greater chest tube drainage, and no specific complication differed significantly [26].

Therefore, what Z0030 tests is whether extending dissection beyond an already rigorous sampling protocol confers therapeutic benefit. It does not test adequate staging against inadequate staging, and it does not test Standard 5.8 against non-compliance. Its implication for the therapeutic model is real but bounded: in a population whose mediastinum has already been systematically sampled to at least the Standard 5.8 level, removing further nodal tissue did not remove disease that mattered for survival or recurrence. That is evidence about extent beyond adequacy. It is not evidence that adequacy itself lacks value, and we do not use it as such. A surgeon reading Z0030 as support for the proposition that once systematic sampling has been performed, complete lymphadenectomy adds little, is reading it correctly.

### 5.3. Standard-Concordant Sampling and Outcome

The staging model is harder to test directly, and the most informative data comes from observational series that must be read with their limitations in view. In a large prospective institutional database from a single high-volume cancer center, examined before the standard came into force, resections concordant with the three-mediastinal and one-hilar criterion had significantly higher rates of nodal upstaging than non-concordant resections, 21% versus 13%. What they did not have was a significant difference in the administration of adjuvant systemic therapy, or in locoregional or distant recurrence, or in overall survival among clinical stage I and II patients; a survival advantage was demonstrable only in clinical stage III [30]. Because baseline nodal evaluation at such a center is likely to be thorough even in the nominally non-concordant group, contamination between the comparison arms would be expected to bias these comparisons toward the null, and the finding should be weighed accordingly. A single-institution comparison of two versus three or more N2 stations in clinical stage I disease, from another high-volume center, reached the same negative conclusion on survival, disease-free survival, and recurrence pattern, subject to the same caveat [29].

The wider evidence identified by the structured search is more balanced than the earlier version of this review conveyed. In the National Society of Thoracic Surgeons database, 34,871 of 52,497 resections between 2021 and 2024 met the standard; concordance was associated with higher nodal upstaging (adjusted odds ratio 1.19, 95% CI 1.12 to 1.27), and there was no difference in major morbidity or mortality [9]; this national finding supersedes the single-institution complication signal we had earlier reported, and we no longer cite that signal. A Danish national registry cohort of 6615 anatomical resections found that adherence to the National Comprehensive Cancer Network sampling recommendation was not associated with overall survival or upstaging after propensity matching [11]. A Mayo Clinic series of 654 clinical stage IA resections found higher upstaging with concordance (21.3% versus 12.5%) and no significant difference in time to recurrence or overall survival, although non-concordant pN0 patients had survival resembling pN1 [12]. Two British single-center series found no overall survival association with IASLC-defined adequacy [17,21], and an early Memphis series found none with guideline-defined good-quality resection [23]; a Polish single-center series of 3215 resections found worse survival with inadequate staging [19]. Against this, a large Veterans Health Administration cohort with manually abstracted operative and pathology data found that station-based adherence was associated with recurrence-free survival while count-based adherence was not, which is a genuine point in favor of the station criterion specifically. The authors were nonetheless explicit that the incremental advantage over the count-based criterion was minimal [4]. A population-based re-enactment of Z0030 found that the survival advantage of mediastinal dissection over less comprehensive staging was confined to teaching institutions, with no significant difference at non-teaching institutions [27]. Table 2 summarizes these studies. Read together, the studies we identified establish that the first link in the staging model, from station sampling to upstaging, is consistently present in national and institutional data alike; that a survival or recurrence association with station-based or lobe-specific adherence has been found in some adjusted cohorts [4,13,31] and not in others [11,12,17,21,29,30]; and that the balance of that evidence, in early-stage disease, raises uncertainty about the survival link rather than resolving it in either direction. That is the extent of what a review of this kind can say.

### 5.4. Stage Migration

Any observed association between thoroughness of nodal examination and stage-specific survival must contend with the Will Rogers phenomenon, described four decades ago in precisely this setting [47]. More thorough examination moves occult-node-positive patients out of the node-negative group and into the node-positive group. The node-negative group loses its worst members and its survival improves. The node-positive group gains its best members and its survival also improves. Both stage-specific curves shift upward with no patient having benefited.

This is not a marginal technical caveat. It is the expected consequence of the intervention the metric measures, and it will generate exactly the association that is used to justify the metric. Registry analyses stratified by pathological stage are structurally vulnerable to it, and the foundational registry studies cited in Section 3 are stratified in exactly this way [34,35]. Analyses of overall survival in the whole cohort are not, which is one reason why the failure to demonstrate an overall survival benefit in early-stage disease in adequately powered contemporary series carries weight, subject to the bias toward the null already noted.

### 5.5. Confounding, Bias Toward the Null, and the Burden of Proof

It would be inconsistent to demand causal evidence from studies that support the metric while accepting observational evidence from studies that do not. We therefore state the evidentiary position explicitly. Observational studies in this field are confounded in both directions. Confounding by indication favors the metric: a surgeon who performs a thorough mediastinal dissection is not sampling at random from the operative population, and the anatomical, physiological, and oncological factors that make a compliant dissection feasible also predict survival independently. The institutional pattern is consistent with this, since a survival advantage that appears in teaching institutions and disappears in non-teaching institutions within the same analysis is more readily explained by correlated processes of care than by a biological mechanism [27]. Contamination and ceiling effects work against the metric: at high-volume centers the non-compliant group is often well-staged, which compresses the difference between arms and biases toward the null. The complication signal in one concordance series illustrated the same problem in the other direction, and the national database has since found no morbidity or mortality difference with concordance [9]; we regard the question of harm as closed and do not raise it against the metric.

The evidentiary asymmetry that remains after these concessions is a matter of burden, not of standard. The party attaching accreditation consequences to a measure bears the burden of demonstrating that the measure is linked to the outcome it is supposed to protect. Observational null findings do not discharge the opposite burden, and we do not claim that they do. Their relevance is narrower: they represent the continued absence of the positive evidence that validation for accountability use would require. The correct conclusion from the available evidence is that the survival link has not been demonstrated in early-stage disease, not that it has been refuted.

### 5.6. Adjuvant Therapy: Delivery Failure or Metric Failure?

The pivotal observation that concordant sampling produced more upstaging without more adjuvant therapy admits two interpretations with opposite policy implications, and both deserve statement. The first is that the staging model fails at its second link. The second is that the metric is doing exactly what it should, finding the patients, and that the failure is downstream in the treatment delivery system. The second reading has considerable support. Adjuvant systemic therapy has historically been delivered to only a fraction of eligible patients: in a national database analysis of 37,571 patients with completely resected stage II to IIIA disease, only 54.9% received adjuvant therapy, with age, comorbidity, and socioeconomic factors independently associated with non-receipt [48], and in a population-based Canadian cohort only 55% of resected patients were seen by a medical oncologist at all [49]; in a complete Danish national registry, among patients with unsuspected nodal upstaging after lobectomy for clinical stage I disease, 60.4% received adjuvant chemotherapy and 38.7% completed all four cycles, with age and comorbidity rather than surgical approach determining compliance [24]. If half of node-positive patients do not receive adjuvant therapy regardless of how they were staged, then upstaging cannot translate into treatment for that half, and the absence of a survival difference is an indictment of delivery rather than of detection.

The two readings can, in principle, be discriminated. Under the delivery-failure reading, the survival benefit of concordant sampling should emerge in the subgroup of upstaged patients who did receive adjuvant therapy and should be absent in those who did not. Under the metric-failure reading, no such interaction is expected. To our knowledge, that stratified analysis has not been published, and it is the single most informative analysis that the existing institutional databases could now provide. Until it is performed, the honest position is that the broken link may lie in the treatment system, in the metric, or in both, and that the policy response differs sharply according to which. Figure 2 has been annotated to reflect the uptake rates at the relevant link.

### 5.7. The Biomarker-Directed and Neoadjuvant Eras: Established Evidence and Surgical Hypotheses

This section separates what randomized trials have established from what follows only as hypothesis, and it presents the reading that most practicing surgeons will find compelling first because, on reflection, we find it the stronger of the two. What is established is this. Adjuvant osimertinib is indicated for resected EGFR-mutated disease from stage IB, on the basis of a hazard ratio for disease recurrence or death of 0.17 in stage II to IIIA disease and a subsequently demonstrated overall survival benefit [50,51]. Adjuvant alectinib is indicated for resected ALK-positive disease from stage IB [52]. Adjuvant atezolizumab and pembrolizumab were tested in populations that began at stage IB or II, with eligibility defined partly by tumor size and PD-L1 expression rather than by nodal status alone [53,54]. Neoadjuvant nivolumab plus chemotherapy improves event-free survival and pathological complete response, and perioperative pembrolizumab does likewise [55,56]. Drug labels remain stage-annotated, and the absolute benefit of adjuvant targeted therapy is largest in node-positive disease.

The first and, in our view, stronger inference from this evidence is that accurate nodal staging has become more consequential, not less. A biomarker-positive patient whose occult N2 disease goes undetected may be denied a therapy of very large effect; a patient with pathological nodal downstaging after neoadjuvant treatment (ypN0) has a materially different prognosis and treatment plan from one without. The incentive to sample thoroughly is greater than it was when the standard was written.

The second inference is a hypothesis, and we label it as such. In each of the adjuvant trials, a proportion of eligibility is determined by T descriptor and biomarker rather than by nodal upstaging, and to that extent the link that nodal staging was supposed to supply may be bypassed for some patients. Where effective targeted therapy exists, the prognostic gap between node-positive and node-negative patients that upstaging was assumed to expose may have narrowed, which would be most pronounced in populations with a high prevalence of EGFR mutation. Whether either effect is large enough to alter the survival association of the metric has not been tested in any dataset we identified, and the rationale for the metric may or may not differ across molecular and geographic populations. We raise the possibility; we do not assert it.

The neoadjuvant setting generates two further surgical hypotheses. Treated mediastinal fields are fibrotic, station boundaries are harder to define, and nodal yield per station may fall for reasons unrelated to surgical effort; conversely, the prognostic weight of ypN status may make thorough sampling more, not less, important. These are plausible clinical observations rather than findings. Neither compliance with Standard 5.8 nor its association with outcome has been characterized after neoadjuvant chemoimmunotherapy in any study we identified, and this is the setting in which validation is most needed and most absent.

## 6. Nodal Adequacy After Sublobar Resection

Three operations are at issue in this section, and they are not variations along a continuum. Lobectomy removes the lobe and its lymphatic drainage en bloc. Anatomical segmentectomy removes a bronchovascular unit with its intersegmental and intraparenchymal nodes, and its lymphatic logic, margin considerations, and expectations of N1 assessment differ from lobectomy in ways that matter for any nodal standard. Wedge resection is non-anatomical, removes no defined drainage territory, and carries different indications altogether. We treat them separately below.

JCOG0802/WJOG4607L randomized 1106 patients with clinical stage IA non-small cell lung cancer of 2 cm or less with a consolidation-to-tumor ratio above 0.5 to segmentectomy or lobectomy, and found five-year overall survival of 94.3% after segmentectomy versus 91.1% after lobectomy, satisfying both non-inferiority and superiority criteria, at the cost of roughly double the rate of local relapse [57]. CALGB/Alliance 140503 randomized 697 patients with peripheral clinical T1aN0 disease to sublobar or lobar resection and found sublobar resection non-inferior for disease-free survival with similar overall survival [58]. Subsequent analyses have refined rather than reversed these conclusions, identifying pure-solid radiological appearance and inadequate margin distance as drivers of locoregional relapse after segmentectomy [59,60].

A point about trial design should be made explicitly because readers familiar with the trials will raise it. CALGB 140503 randomized only after intraoperative confirmation of node-negative status by hilar and mediastinal sampling with frozen section, and JCOG0802 mandated nodal dissection in both arms. Nodal assessment was embedded in the designs. The non-inferiority of sublobar resection was therefore demonstrated in patients whose nodes had been rigorously assessed, and rigorous nodal evaluation is, on this reading, a prerequisite of the sublobar era rather than a lobectomy-era anachronism within it. We accept this reading entirely. It does not weaken the case for procedure-specific standards; it strengthens it because it means that the nodal assessment which underwrites sublobar resection needs to be specified in terms appropriate to the operation.

Standard 5.8 applies to all of these operations. Compliance with it does not follow. In a multicenter cohort of early-stage resections, 43% of lobectomy patients had three mediastinal and one hilar station sampled, against 23% of sublobar resection patients [31]; in a Mayo Clinic series of clinical stage IA resections the standard was met in 51.6% of lobectomies, 29.9% of segmentectomies, and 17.1% of wedge resections [12]; and in the national Society of Thoracic Surgeons database, segmentectomy (adjusted odds ratio 2.67) and lobectomy (3.75) were each far more likely than wedge resection to be concordant, which places anatomical segmentectomy much closer to lobectomy than to wedge [9]. National benchmarking of segmentectomy for clinical stage I disease shows nodal upstaging rates of approximately 6%, roughly half those typically reported after lobectomy, with no significant difference across open, video-assisted, and robotic approaches [61]. For wedge resection, the position is more extreme, with a national database analysis of resections performed between 2010 and 2014 reporting that a substantial proportion of wedge resections had no nodes examined at all [62]; practice has changed since, and the figure should be read as historical.

The interpretation of low compliance after sublobar resection admits two readings, and the evidence we identified now favors one of them. On one reading, a standard written for lobectomy is being applied to operations for which its anatomical logic does not transfer, and low compliance partly reflects that mismatch. On the other reading, nodal assessment after sublobar resection is frequently inadequate, and the compliance gap is precisely why a minimum standard is needed. The second reading has more support than we earlier allowed. After sublobar resection, Standard 5.8 concordance was associated with reduced one-year recurrence, adjusted hazard ratio 0.62 (95% CI 0.39 to 0.98), while no association was found after lobectomy [31]. In 27,517 National Cancer Database sublobar resections for tumors of 20 mm or less, examination of ten or more nodes was independently associated with survival, in segmentectomy and wedge resection alike [16]. And in a national analysis of 11,006 segmentectomies, only 12.6% met a composite of adequate nodal sampling, negative margins, and appropriate tumor size; adherence was more likely at academic programs, and adherence to all measures was associated with survival (hazard ratio 0.67, 95% CI 0.56 to 0.79) [15]. The consistent picture across these three datasets is that nodal assessment after sublobar resection is frequently inadequate, that inadequacy is associated with worse outcomes, and that the standard identifies it. A practicing surgeon who performs segmentectomy does not regard nodal assessment as less important because less lung has been removed; the oncological justification for preserving parenchyma rests on proving N0 disease, and the trials that established segmentectomy embedded nodal evaluation in their designs for that reason. Low Standard 5.8 compliance after segmentectomy therefore most plausibly identifies a real contemporary quality problem, and we say so in the Conclusions.

What follows from this is not exemption but attention, and possibly specification. Two points remain, and we frame both more modestly than before. The first is that compliance should be reported stratified by resection type rather than adjusted for it. Adjusting for resection extent would risk normalizing precisely the behavior the standard exists to detect; stratified reporting preserves the signal while allowing programs to see where the deficit lies. The second is a research hypothesis, not a proposal for replacement. The current criterion says nothing about the intersegmental and intraparenchymal stations (11, 12, and 13) that carry the first-echelon drainage of a segment. A segment-specific specification, built around the segmental and interlobar N1 stations along the drainage of the resected segment together with the lobe-specific mediastinal stations, is anatomically attractive and has indirect support from the observation that lobe-specific mediastinal sampling was associated with lower recurrence and higher upstaging in a manually abstracted national cohort [13]. It has not been validated in any form; retrieving all such stations is not always feasible in a thoracoscopic segmentectomy, and we would be inconsistent to criticize one unvalidated threshold and then propose another as its obvious successor. We present it as the hypothesis that a prospective evaluation of nodal standards in segmentectomy should test, and nothing more.

## 7. Alternative Measures and Changing Context

Criticism of a metric is only useful if the alternatives are subjected to the same scrutiny. They should be, and Table 3 applies the same criteria to each.

Station-based criteria are the incumbent, and their advantage over counts is real but modest [4]. Lymph node ratio carries consistent prognostic information, with a meta-analysis of 20 cohorts and 76,929 patients confirming an association between lower ratio and better overall, disease-free, and cancer-specific survival [63]. Its limitation as a quality metric is structural: the ratio is undefined when no nodes are positive, which describes the majority of curative-intent resections. The log odds of positive nodes (LODDS) incorporates a continuity correction and is therefore defined in node-negative patients, and in a registry of 17,369 radically resected patients it discriminated survival more finely than either pN category or ratio [64], with comparable findings in node-positive SEER cohorts [65]. Both, however, are prognostic indices computed after the fact rather than process targets a surgeon can aim at, and neither has been tested as an accountability instrument.

Specimen-level process interventions are, on the evidence reviewed above, the most effective lever currently available [28,41]. This makes them excellent quality improvement tools. It also means that when they are deployed, the resulting compliance figure measures the pathway rather than the surgeon, which is the correct level for institutional accountability. National collaborative approaches raised the proportion of participating programs meeting the 80% threshold from 40.7% to 67.2%, with the largest gains in community programs [6], and structured multidisciplinary initiatives at individual health systems have produced comparable improvements [66]. These are genuine achievements in standardizing documentation and specimen handling. Whether they have improved survival is a separate question that the compliance literature does not address. A resource dimension should also be recorded: systematic station sampling costs operative time, and thorough grossing costs pathologist time, and any revision of the standard will redistribute those costs rather than eliminate them.

Composite outcome measures offer a different route. Textbook outcome for lung cancer resection bundles margin-negative resection, adequate nodal assessment, acceptable length of stay, freedom from mortality and readmission, and receipt of indicated adjuvant therapy into a single binary endpoint, achieved in roughly one quarter of patients and independently associated with survival [67]. Its relevance here is diagnostic as much as prescriptive: failure to achieve the composite was driven predominantly by inadequate nodal assessment, which means the composite largely reproduces the nodal metric while obscuring that it has done so. Composite measures inherit the validity problems of their weakest component.

Two developments outside the operating room alter the context in which the metric operates. Neither is an alternative to intraoperative nodal assessment, and neither makes its quality irrelevant; both are complementary. The first is preoperative endosonographic staging. In a randomized non-inferiority trial of 360 patients with an indication for mediastinal staging after negative systematic endosonography, immediate resection produced an unforeseen N2 rate of 8.8% against 7.7% after confirmatory mediastinoscopy [68], and endobronchial ultrasound has displaced mediastinoscopy as the standard of care in much of the world [69]. As the residual probability of occult mediastinal disease at resection falls, the marginal yield of intraoperative station sampling falls with it, and a fixed threshold calibrated to an earlier staging era becomes less well-matched to the population it is applied to. The second is molecular residual disease assessment. Longitudinal circulating tumor DNA analysis after resection identifies patients at high risk of recurrence and predicts adjuvant chemotherapy benefit in stage II and III disease [70]. Its landmark sensitivity remains imperfect, serial testing is not standard of care, and pathological nodal status will remain required for trial eligibility, radiotherapy planning, and prognosis for the foreseeable future. Its relevance to the present argument is only that the question a nodal metric answers, whether residual disease is likely, will increasingly be answered by more than one method, and a quality framework anchored solely to nodal stations should expect its share of that answer to change.

## 8. Evaluating the Metric Against an Established Framework

### 8.1. Anchoring the Criteria

In an earlier version of this review, we proposed four requirements for a defensible nodal quality metric without stating where they came from. They are not novel and should not be presented as such. Donabedian’s framework distinguishes structure, process, and outcome, and holds that a process measure earns its place through a demonstrated causal linkage to outcome [71]; Birkmeyer and colleagues applied that framework to surgery specifically and noted that process measures are attractive precisely because they are actionable and require little case-mix adjustment, provided the linkage to outcome is established [72]. The National Quality Forum’s measure evaluation criteria, which govern endorsement of measures for accountability use in the United States, require importance (including evidence linking the measured process to outcome, criterion 1c), scientific acceptability of measure properties (reliability and validity, criterion 2c, including risk or case-mix adjustment where performance is affected by factors outside the accountable party’s control, criterion 2e), feasibility, and usability across the settings in which the measure will be applied [73]. Our four requirements map onto these without strain, and we restate them in those terms.

### 8.2. The Four Criteria Restated

First, appropriate handling of case mix (NQF 2e). Not every variable that influences compliance is a legitimate adjustment variable because risk adjustment can adjust away poor quality. The variables shown to affect concordance fall into two groups. Non-modifiable case mix, which a surgeon cannot alter at the time of resection, includes histology, laterality, prior treatment, calcified or fibrotic nodal fields, and patient age and comorbidity [9,10,32]; these are candidates for adjustment. Resection extent and operative approach are different: they are chosen, they may themselves mediate quality of care, and adjusting for them could normalize the behavior the metric is meant to detect. For these, the defensible approach is stratified reporting, not adjustment, so that a program can see its compliance separately for lobectomy, segmentectomy, and wedge resection without any of the three being excused. Standard 5.8 currently applies neither adjustment for the first group nor stratification for the second, and that, rather than any absolute deficiency, is the criterion 2e gap we identify.

Second, attribution to the accountable party (NQF 2c, validity). The metric should measure something the accountable party controls. At the institutional level, Standard 5.8 meets this because institutions control the whole pathway, and that is the level at which its formal consequences apply (Section At Which Level Is Standard 5.8 Actually Used). At the level of individual technical performance, the evidence we identified does not support it because specimen-handling interventions that require no change in operative technique move it further than surgeon-directed education does [28], and because a single adjusted analysis found platform to be independently associated with concordance [10]. At the intermediate level of surgeon-led process performance, the metric is more defensible, since the surgeon owns much of that pathway, and this is the level at which collaborative surgeon-level feedback is in fact used.

Third, demonstrated linkage to a patient-centered outcome (NQF 1c), under a proportionate evidentiary standard, with the proximal outcome given its due weight. The immediate purpose of nodal assessment is accurate pathological staging, and on that outcome the metric is consistently validated: concordance increases upstaging in every dataset we identified, including the national database [9]. Overall survival is several causal steps downstream and is affected by tumor biology, systemic treatment, comorbidity, and uptake, and we now give it less conceptual weight as the decisive validation test than the earlier version of this review did. Randomized evidence is not the bar, and if it were, most surgical process measures in current use would fail it; the SCIP precedent shows what happens when process measures are adopted without that linkage and then found not to predict the outcome they were meant to protect [3]. The NQF standard is a body of evidence, which may be observational, demonstrating that the process is associated with the outcome after adjustment. For Standard 5.8 that body of evidence is currently mixed: upstaging is consistently increased; recurrence-free survival was associated with station-based adherence in one national cohort [4] and recurrence with lobe-specific adherence in another [13]; one-year recurrence was reduced after sublobar resection in a multicenter series [31]; survival was associated with composite quality measures that include nodal sampling in national data [14,15,22]; and overall survival was not different with concordance in a Danish national cohort [11], in the two largest institutional series [29,30], or in three further single-center series [12,17,21], several of which are subject to bias toward the null. A staging-completeness measure might reasonably be validated against a validated intermediate such as pathological nodal upstaging rather than against survival, provided the intermediate’s own linkage to outcome is established; whether it is, in the biomarker era, is exactly the question Section 5.7 leaves open.

Fourth, applicability across resection types (NQF usability). A metric applied to lobectomy, segmentectomy, and wedge resection should be specified so that it means the same thing in each. Standard 5.8 is not, and Section 6 sets out what specification would require.

### 8.3. Floor Versus Discrimination

Accountability metrics serve two functions that the four criteria weigh differently. The first is to eliminate the left tail of performance, and the second is to discriminate among competent programs. A binary minimum standard is well-suited to the first function precisely because it is insensitive to degree: it identifies the wedge resection with no nodes examined, the lobectomy with no mediastinal station reported, and the program in which such cases are common. Much of the critique above bears on the second function and not the first. On our own analysis, Standard 5.8 retains clear value as a floor. The compliance literature shows that the floor was not being met by a large minority of programs before the standard was introduced [5], that it can be raised by pathway interventions [6,28], and that the cases it identifies are ones in which staging was incomplete by any definition. The four criteria apply with full force only when the same binary value is used to discriminate among programs or surgeons who are all above the floor, and that is the use we argue is not yet validated.

### 8.4. What Follows, and What Does Not

The conclusion these criteria support is narrower than the one an earlier version of this review drew, and we state it in full, in language proportionate to a narrative review. Standard 5.8 is well-supported as an institutional minimum standard for staging completeness. It has raised the floor, it identifies genuinely incomplete staging, it increases nodal upstaging without a morbidity penalty in national data, and the collaborative work has raised adherence to it is valuable work. The available evidence identified in this review raises uncertainty regarding its extension to individual-surgeon benchmarking because the value reflects surgeon-led process and institutional pathway as much as individual technique; we did not identify convincing validation for that extension, and it is not part of the standard’s formal use. It does not currently distinguish non-modifiable case mix, which could be adjusted, from resection extent and approach, which should be reported stratified. And the low compliance observed after sublobar resection most plausibly identifies a real quality problem that the standard is right to detect, while a segment-specific specification remains a hypothesis for prospective testing. Each of these is a specific and modest observation, and none of them is an argument against nodal evaluation.

The constructive path has three elements: compliance should be reported stratified by resection type, and a segment-specific nodal specification should be evaluated prospectively before it is proposed as a standard; reporting should distinguish the surgical, pathological, and specimen-handling contributions rather than aggregating them, so that improvement effort can be directed where the deficiency lies; and the outcome question should be addressed by the stratified analysis described in Section 5.6 and, ideally, prospectively, and by a systematic review with a formal risk-of-bias assessment across multiple databases, which this narrative review is not and which the evidence base now justifies.

### 8.5. Clinical Implications

Because a critique of a quality metric can be misread as a critique of the practice it measures, we state the clinical implications directly, and we address them to trainees and to surgeons outside high-volume centers in particular. Nothing in this review reduces the obligation to perform systematic nodal assessment at every curative-intent resection. Thorough hilar and mediastinal nodal evaluation remains the foundation of accurate pathological staging, of prognosis, of the determination of complete resection, and of treatment planning, and it is more consequential, not less, now that effective adjuvant and neoadjuvant therapies exist for patients whose nodal disease is found. The compliance shortfall documented in this review, particularly after sublobar resection, describes real gaps in staging that should be closed. The recommendations we make are directed at those who design, adjust, and attach consequences to the metric, not at the surgeon in the operating room. For the surgeon, the correct reading of this review is that Standard 5.8 should be met, that meeting it requires ownership of the whole specimen pathway, and that the standard’s own specification for segmentectomy needs to be improved rather than relaxed. A surgeon who reads it as permission to do less has read it incorrectly, and we would regard that outcome as a failure of the review.

## 9. Limitations

This is a narrative rather than a systematic review, and Section 2 describes a reproducible search, its yield, and the screening outcome so that the reader can judge what was and was not identified; only one database was searched, no validated risk-of-bias instrument exists for this study type, and none was applied, and the selection nonetheless reflects the authors’ judgment of relevance, so the review cannot claim to characterize the totality of the validation evidence and its conclusions are framed accordingly. Several of the pivotal negative studies are single-institution series from high-volume centers, where baseline nodal evaluation is likely to be thorough even in the non-compliant group, which would attenuate any true effect of the standard; we have flagged this at each point of citation and treated the resulting bias toward the null as a limitation of the evidence against the metric, not as a point in its favor. Much of the compliance literature postdates implementation of Standard 5.8 by only a few years, so long-term outcome data under the standard are not yet available, and no data exist on its behavior after neoadjuvant therapy. The context-changing developments discussed in Section 7 are at differing stages of validation, and none have been tested as an accountability instrument. We should also record our own position. Several of the present authors, including the corresponding author, wrote the earlier synthesis of staging practice and compliance strategy that this review interrogates [7]; that synthesis took the validity of the standard for its stated purpose as given, and the present review is in part a re-examination of that assumption by its own authors. Readers are entitled to weigh that. Finally, we have concentrated on the North American accreditation framework because it is the setting in which nodal adequacy carries formal consequences; the argument may transfer imperfectly to systems organized around different definitions of adequate lymphadenectomy, and, as Section 5.7 notes, the rationale for the metric may differ materially in populations with different molecular epidemiology.

## 10. Conclusions

Nodal evaluation in lung cancer surgery is important, and the case for evaluating nodes thoroughly is not in dispute. What this review has examined is the inferential weight placed on a particular threshold when it is used for accountability, and the conclusions that follow are framed as what the evidence we identified does and does not support, not as verdicts on the totality of the literature. Judged against the National Quality Forum criteria, Standard 5.8 is well-supported as an institutional minimum standard for staging completeness: it reliably increases nodal upstaging, including in a national database of over 52,000 resections without a morbidity penalty; it identifies incomplete staging, and adherence to it can be raised by pathway interventions. Its formal use is institutional, and at that level we identified nothing that undermines it. Its extension to individual-surgeon benchmarking, which is emerging informally in quality collaboratives, is a use for which we did not identify convincing validation because the reported value reflects surgeon-led process and institutional pathway as well as individual technique; the available evidence raises uncertainty about that extension rather than refuting it. The standard does not currently distinguish non-modifiable case mix, which could be adjusted, from resection extent and approach, which should be reported stratified rather than adjusted away. The survival link that would license its use as an outcome-validated measure has been found in some adjusted cohorts and not in others, and remains uncertain in early-stage disease.

The sublobar evidence deserves its own sentence because it changed our conclusion. Low Standard 5.8 compliance after segmentectomy and wedge resection is associated with worse outcomes in national and multicenter data, and most plausibly identifies a real contemporary quality problem that the standard is right to detect. A segment-specific specification is a hypothesis worth testing prospectively, but it is not a replacement we are in a position to propose. None of this argues for less nodal evaluation. It argues for precision about what the metric has been validated to measure, at what level of attribution, and for which operations, so that a standard that is doing valuable work as a floor is not asked to carry inferences it cannot yet bear. The accreditation consequences attached to it should be read as a statement about the degree of inference currently placed on the metric, and the proportionate response is stratified reporting, adjustment for non-modifiable case mix, the stratified adjuvant analysis that would clarify the outcome question, and a formal systematic review, not abandonment.

## Figures and Tables

**Figure 1:**
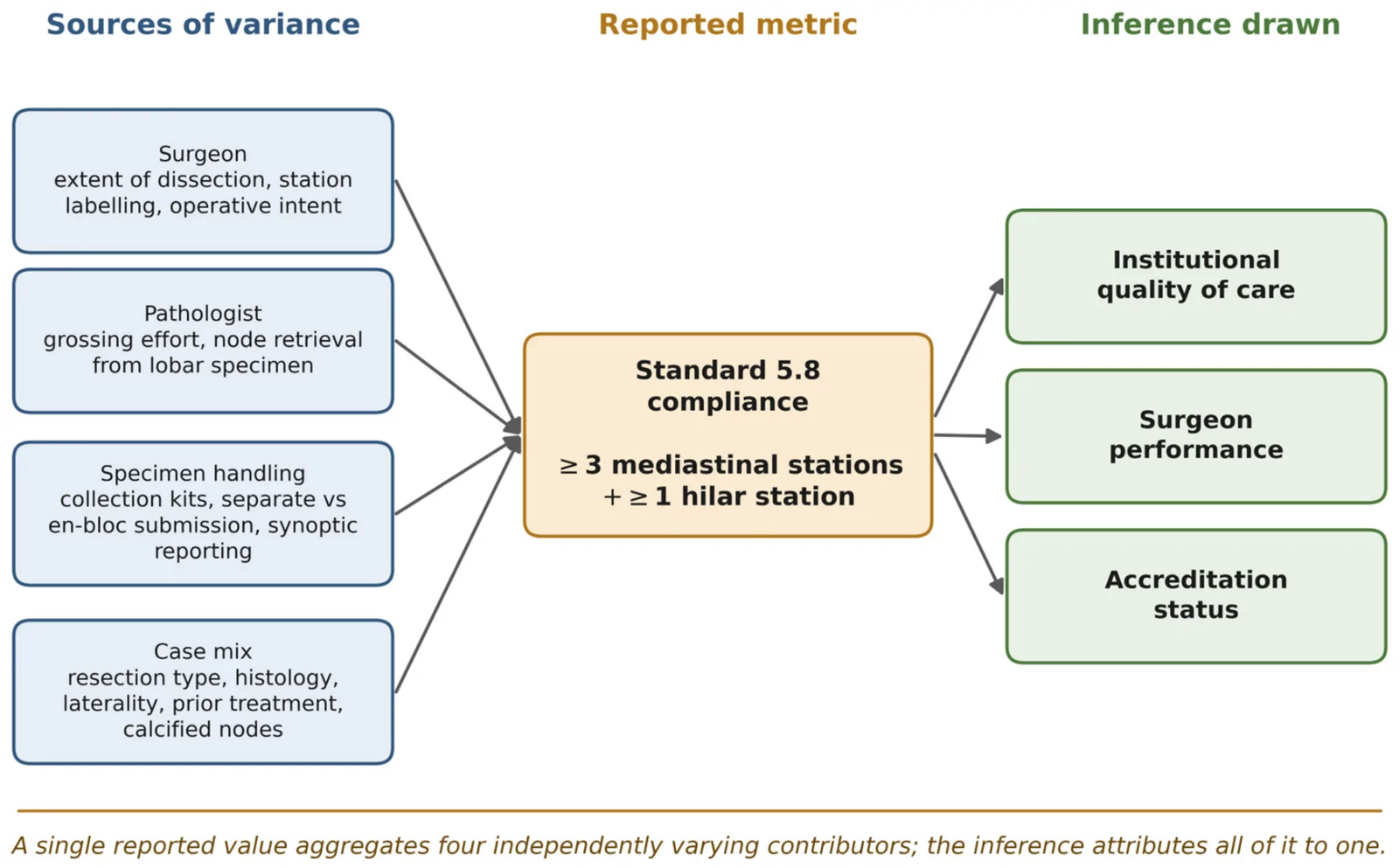
The attribution problem in nodal adequacy measurement. Independently varying contributors are aggregated into a single reported value, from which inferences are drawn about a single accountable party. Interventions acting on the specimen-handling pathway alone can shift measured compliance by more than fourfold without any change in operative technique. The figure bears on the level at which the metric can fairly be attributed, not on its legitimacy as a measure of the nodal-assessment pathway.

**Figure 2:**
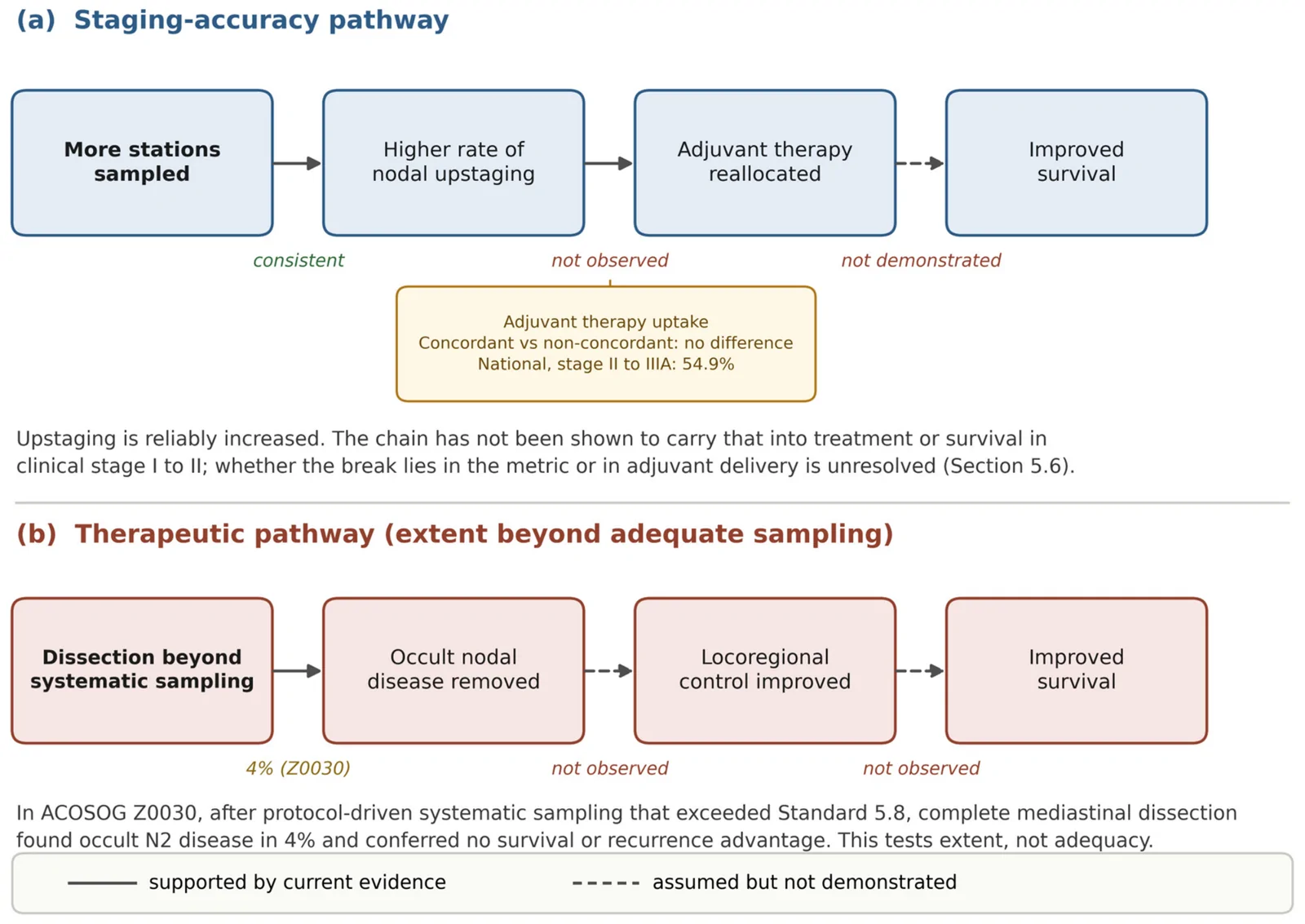
Two causal models linking nodal evaluation to survival, with the current evidence status of each link. (**a**) The staging-accuracy pathway, in which benefit is mediated by upstaging and adjuvant therapy reallocation; the link from upstaging to adjuvant therapy is annotated with the adjuvant therapy uptake observed in the concordance series and nationally. (**b**) The therapeutic pathway, in which benefit is intrinsic to removal of occult disease; the evidence applies to an extent beyond adequate sampling. The solid arrows denote links supported by current evidence; the dashed arrows denote links that are assumed but not demonstrated.

**Table 1:** Frameworks for defining nodal adequacy in curative-intent lung cancer resection, with the unit of adequacy each employs and its principal limitation when used as an accountability measure. CoC, Commission on Cancer; IASLC, International Association for the Study of Lung Cancer; LODDS, log odds of positive lymph nodes; NCCN, National Comprehensive Cancer Network.

Framework	Operational Criterion	Unit of Adequacy	Principal Limitation as an Accountability Measure
CoC surveillance metric (historical)	At least 10 lymph nodes examined from anywhere in the chest	Node count	Anatomically indifferent; satisfied by intrapulmonary nodes without any mediastinal assessment; heavily dependent on pathologist grossing effort
CoC Operative Standard 5.8	Nodes reported from at least 3 distinct mediastinal (N2) stations and at least 1 hilar (N1) station, documented synoptically	Station count	Written for lobectomy but applied to all curative-intent resections; treats all stations as interchangeable; binary and therefore insensitive to degree
NCCN resection quality criteria	Anatomic resection, negative margins, hilar or intrapulmonary node examination, and at least three mediastinal stations examined	Composite of station count and resection attributes	Bundles nodal evaluation with margin and resection-extent components, so a single failure rate conceals which domain failed
IASLC complete resection definition	Free margins, systematic or lobe-specific systematic nodal dissection, no extracapsular extension, and negative highest mediastinal node	Procedural completeness, with an explicit uncertain-resection category	Requires operative and pathological detail rarely captured in registries; the uncertain category is difficult to audit at scale
Lymph node ratio	Positive nodes divided by nodes examined	Nodal disease burden relative to sampling effort	Undefined in node-negative disease, which is the majority of resections; prognostic rather than actionable
Log odds of positive nodes (LODDS)	Log of (positive nodes plus 0.5) divided by (negative nodes plus 0.5)	Nodal disease burden with a continuity correction	Defined in node-negative patients, unlike the ratio; retains prognostic rather than process character and has not been tested as an accountability instrument

**Table 2:** Studies bearing on the validity of nodal adequacy as a quality metric, ordered by publication year and selected according to the criteria in Section 2, with a structured appraisal of threats to validity and their likely direction. Patient numbers are those in the analytic cohort. ACOSOG, American College of Surgeons Oncology Group; aHR, adjusted hazard ratio; aOR, adjusted odds ratio; CI, confidence interval; cStage, clinical stage; DFS, disease-free survival; HR, hazard ratio; IASLC, International Association for the Study of Lung Cancer; N1, hilar nodal station; N2, mediastinal nodal station; NCCN, National Comprehensive Cancer Network; NCDB, National Cancer Database; OS, overall survival; RFS, recurrence-free survival; STS, Society of Thoracic Surgeons; UICC, Union for International Cancer Control; VA, Veterans Affairs.

Study	Design and Setting	Patients	Nodal Criterion	Principal Finding	Structured Appraisal: Threats to Validity and Likely Direction
Darling 2011 (ACOSOG Z0030) [25]	Multicenter randomized trial	1023	Complete dissection vs systematic sampling in patients already staged by sampling	No difference in OS, DFS, or recurrence; occult N2 in 21 dissection patients	Randomized; tests extent beyond adequate sampling, not adequate vs inadequate staging. Not applicable to Standard 5.8 compliance
Allen 2010 [26]	Single metropolitan area, retrospective	746	Guideline-defined good-quality resection (NCCN, RADIANT, ACOSOG criteria)	No survival difference; inadequate mediastinal sampling the commonest deficit	Early era, unadjusted survival comparison; small numbers meeting criteria. Bias toward the null
Samson 2016 [22]	NCDB, retrospective	133,366	Composite of four measures incl. 10 or more nodes	Meeting all four measures associated with OS (HR 0.39)	Composite; cannot isolate nodal component; stage-stratified registry exposed to stage migration; residual confounding by institution. Favors the metric
Edwards 2017 [21]	Single UK center, retrospective	1301	IASLC 3-station adequacy	No OS difference; worse survival if pN2 and inadequate	Single center; adequacy improved 14% to 53% over period, so comparison partly temporal. Bias toward the null
Pawelczyk 2018 [19]	Single Polish center, retrospective	3215	UICC 6 nodes/stations	Inadequate staging associated with worse survival across stages	Single center; stage-stratified comparison exposed to stage migration; adjustment limited. Favors the metric
Bosch 2018 [20]	Single institution plus SEER	888 and 10,856	10 or more nodes	No OS difference 1 to 9 vs 10 or more; zero nodes worse	Count-based; registry adjustment limited. Supports floor, not threshold
Ray 2020 [27]	Population-based, four US regions	1942	Dissection vs sampling vs neither	Survival advantage for dissection at teaching institutions only	Observational; institution-level effect modification suggests co-process confounding. Favors the metric where positive
Dezube 2020 [18]	NCDB, retrospective	102,225	Node count as continuous variable	Survival gain concentrated at 0 to 5 nodes; no support for 10-node threshold	Registry; stage migration; adjustment limited. Supports floor, not threshold
Ray 2021 [28]	Population-based stepped-wedge	3734	Collection kit vs heightened awareness vs baseline	CoC attainment 14% to 77% with kit; survival higher with both interventions	Non-randomized stepped wedge; temporal confounding; survival comparison across eras. Favors intervention
Taylor 2021 [17]	Single UK center, retrospective	2380	IASLC 2009 adequacy	No OS association (adjusted HR 0.84, 95% CI 0.64 to 1.09)	Single center; adequacy rose 45% to 85% over period; adjusted. Bias toward the null
Heiden 2022 [4]	VA national, manually abstracted	9749	Count-based vs station-based (3 N2 and 1 N1)	Station-based adherence associated with RFS (aHR 0.82); count-based not	Manual abstraction is a strength; adjusted; contemporaneous comparison. Moderate confounding by indication. Favors the metric
Logan 2022 [15]	NCDB, retrospective	11,006 segmentectomies	Composite: 10 or more nodes, R0, size 2 cm or less	12.6% adherent; adherence associated with OS (HR 0.67)	Composite; cannot isolate nodal component; propensity matched; segmentectomy-specific. Favors the metric in segmentectomy
Takamori 2022 [16]	NCDB, retrospective	27,517 sublobar	10 or more nodes in sublobar resection	10 or more nodes associated with OS in segmentectomy and wedge	Count-based; registry; stage migration. Favors nodal assessment in sublobar resection
Heiden 2023 [14](VALCAN-O)	VA plus NCDB validation	9628 and 107,674	Composite score incl. adequate sampling	Higher score associated with OS and RFS stepwise	Composite; nodal component not isolated; externally validated. Favors composite measures
Subramanian 2023 [13]	VA national, manually abstracted	6201 upper-lobe	Lobe-specific vs station-based vs count-based	Lobe-specific adherence associated with lower recurrence (aHR 0.83) and higher upstaging; not OS	Adjusted; low adherence (7.8% to 23.2%) limits power; competing-risks model. Favors lobe-specific specification
Al-Thani 2024 [29]	Single high-volume US center	581	2 vs 3 or more N2 stations	No difference in OS, DFS, or recurrence, cStage I	Single high-volume center; contamination between arms likely. Bias toward the null
Rocco 2024 [12]	Single high-volume US center	654 cStage IA	Standard 5.8 concordance	Upstaging 21.3% vs 12.5%; OS aHR 0.78 (0.58 to 1.05); non-concordant pN0 resembled pN1	Single center; adjusted; underpowered for survival. Bias toward the null
Resio 2025 [30]	Single high-volume US center, prospective database	9289	Standard 5.8 concordance	Upstaging 21% vs 13%; no difference in adjuvant therapy, recurrence, or OS in cStage I to II; OS benefit in cStage III	Single center; adjusted; nominally non-concordant group likely well-staged. Bias toward the null
Arnarsson 2025 [11]	Danish national registry	6615	NCCN sampling adherence	No OS or upstaging association after propensity matching	National; propensity matched; imputed data; NCCN rather than 5.8 criterion. Bias toward the null if adherence measured imprecisely
Williams 2025 [10]	Statewide collaborative, 43 surgeons	3031	Standard 5.8 concordance	Concordance 77.8%; associated with surgeon volume, resection type, and platform; no upstaging or complication difference	Adjusted for surgeon volume and procedure; surgeon-level; single state. Informative for attribution, not outcome
Baskin 2025 [31]	Multicenter integrated health system	2482	Standard 5.8 concordance by resection extent	No association after lobectomy; reduced 1-year recurrence after sublobar (aHR 0.62, 0.39 to 0.98)	Adjusted; short endpoint; sublobar subgroup small. Favors the metric in sublobar resection; awaits replication
Krantz 2026 [9]	STS national database	52,497	Standard 5.8 concordance	66.4% concordant; upstaging aOR 1.19 (1.12 to 1.27); no morbidity or mortality difference	National; adjusted; no survival endpoint yet; registry variables. Validates upstaging and absence of harm

**Table 3:** Candidate nodal quality measures evaluated against the four requirements of Section 8.2, each mapped to the corresponding National Quality Forum (NQF) criterion. cStage, clinical stage; ctDNA, circulating tumor DNA; LODDS, log odds of positive lymph nodes; MRD, molecular residual disease; N1, hilar nodal station; N2, mediastinal nodal station.

Candidate Measure	Case-Mix Adjustment (NQF 2e)	Attribution to Accountable Party (NQF 2c Validity)	Linkage to Patient-Centered Outcome (NQF 1c)	Applicability Across Resection Types (NQF Usability)	Overall Status
Node count (10 or more)	None applied	Weak: dominated by pathologist and specimen handling	Registry associations only; stage migration unaddressed	Applied uniformly; not specified by procedure	Superseded
Standard 5.8 (3 N2 and 1 N1)	No adjustment for non-modifiable case mix and no stratified reporting by resection type; both are feasible	Institutional: supported, and this is its formal use. Surgeon-led process: defensible. Individual technique: evidence identified does not support benchmarking	Upstaging: consistently validated, including nationally. Survival in cStage I to II: mixed across adjusted cohorts; uncertain. cStage III: supported	Applied uniformly; compliance far lower after wedge than segmentectomy; low sublobar compliance is associated with worse outcome and likely reflects a real quality deficit	Supported as an institutional floor and staging-completeness measure; extension to individual-surgeon benchmarking not supported by evidence identified
Lymph node ratio	Partial: normalizes for nodes examined	Not attributable to a single party	Prognostic association established	Undefined in node-negative disease	Prognostic index, not a process metric
LODDS	Partial: normalizes for nodes examined	Not attributable to a single party	Prognostic association established	Defined in node-negative disease; not tested as a metric	Prognostic index, not a process metric
Specimen-level process measures (collection kits)	Not applicable	Attributable to the specimen pathway; not to the surgeon	Improves measured compliance; outcome effect not established	Applicable to all resections	Effective quality-improvement lever; not an accountability metric
Textbook outcome	Partial, depending on component	Composite; inherits the attribution problems of its components	Associated with survival	Applicable, but driven predominantly by the nodal component	Composite; obscures which component failed
ctDNA/MRD	Not applicable	Not a surgical process measure	Prognostic; validation for accountability absent	Applicable, but measures residual disease rather than staging effort	Complementary to, not a substitute for, nodal staging

## Data Availability

No new data were created or analyzed in this study. Data sharing is not applicable to this article.

## Abbreviations

The following abbreviations are used in this manuscript:

ACOSOG	American College of Surgeons Oncology Group
CoC	Commission on Cancer
cStage	Clinical Stage
ctDNA	Circulating Tumor DNA
IASLC	International Association for the Study of Lung Cancer
LODDS	Log Odds of Positive Lymph Nodes
MRD	Molecular Residual Disease
N1	Hilar Lymph Node Station
N2	Mediastinal Lymph Node Station
NCCN	National Comprehensive Cancer Network
NQF	National Quality Forum
NSCLC	Non-Small Cell Lung Cancer
RATS	Robot-Assisted Thoracic Surgery
SCIP	Surgical Care Improvement Project
VATS	Video-Assisted Thoracic Surgery
